# THE INTERPLAY BETWEEN MEMORY SPECIFICITY AND COGNITIVE INHIBITION

**DOI:** 10.1093/geroni/igac059.2063

**Published:** 2022-12-20

**Authors:** Jessie Chien, Teal Eich

**Affiliations:** University of Southern California, Los Angeles, California, United States; University of Southern California, Los Angeles, California, United States

## Abstract

Pattern separation studies suggest age-related declines in discriminating between a newly and a previously encountered overlapping mental representations. However, when similar experiences occur contemporaneously, shared features may serve as intrinsic factors that interfere with the ability to encode and retrieve events as distinct memories. If so, successful inhibition of competitors might also be necessary to maintain memory specificity. Here, we investigated the interplay between memory specificity and inhibitory control in younger (20-35;n=34) and older (65+;n=37) adults by testing whether and how presenting a target item with a lure item (varied in its similarity to the target) influenced recognition accuracy, probed using a 3-item (target, lure, and a non-presented control) forced-choice recognition test. Surprisingly, we found no age-related differences, including in target accuracy, which was high in both groups (young=80.25%; older=77.86%). While not age-specific, we did find that the pattern of errors varied as a function of the level of similarity between the items at encoding. When target and lure were highly similar, a greater proportion of control items were falsely-recognized relative to lure items. It is possible that inhibition of the lure spread to the target, leaving both inaccessible at retrieval (e.g., a retrieval-induced forgetting effect). When the target and lure were less similar, participants false alarmed more to lure than control, potentially because the lure provided less competition and thus did not necessitate inhibitory control. These results shed light on how inhibitory processes might be differentially involved, but age-invariant, depending on the similarity of encoded items.

